# The Effect of Hemicellulose and Lignin on Properties of Polysaccharides in *Lentinus edodes* and Their Antioxidant Evaluation

**DOI:** 10.3390/molecules24091834

**Published:** 2019-05-13

**Authors:** Feifei Wu, Xin Jia, Lijun Yin, Yongqiang Cheng, Yuxin Miao, Xiuqing Zhang

**Affiliations:** College of Food Science and Nutritional Engineering, China Agricultural University, P. O. Box 40, No.17 Qinghuadonglu, Haidian, Beijing 100083, China; feifeiwu@cau.edu.cn (F.W.); xinjia@cau.edu.cn (X.J.); lyjin@cau.edu.cn (L.Y.); chengyq@cau.edu.cn (Y.C.); miaoyuxin@cau.edu.cn (Y.M.)

**Keywords:** *Lentinus edodes*, polysaccharide, hemicellulose, lignin, antioxidant activity, *Caenorhabditis elegans*

## Abstract

*Lentinus edodes*, whose polysaccharides possess diverse bioactivities, commonly grows on hardwood sawdust composed of hemicellulose, lignin and cellulose. In this study the effect of hemicellulose and lignin on the growth of mycelia, as well as the physicochemical properties of polysaccharides from *L. edodes* mycelia (LEPs) were investigated. The antioxidant properties of LEPs were evaluated through radical scavenging assays in vitro and through the *Caenorhabditis elegans* model in vivo. The results showed that hemicellulose at a concentration of 4% increased the yield of the mycelia biomass to twice that of the control group. Meanwhile, when cultured with 4.0% hemicellulose, the polysaccharide content of the mycelia was raised by 112.2%. In addition, the appropriate concentration of lignin could stimulate mycelia growth and polysaccharide biosynthesis in *L. edodes*. Monosaccharide composition analysis showed that a higher content of xylose was found when mycelia were cultured with higher concentrations of hemicellulose. The molecular structure, including the molecular weight distribution and configuration type, was affected by hemicellulose and lignin. Antioxidant assays indicated that LEPs supplemented with hemicellulose and/or lignin possessed higher radical scavenging abilities in vitro and exhibited a thermal resistance effect on *C. elegans*, implying that the antioxidant effect is potent in vivo. In summary, the addition of hemicellulose and lignin improved the biosynthesis and bioactivity of LEPs.

## 1. Introduction

Polysaccharides are natural biomacromolecules that widely exist in animals, plants and microorganisms. In recent years, fungi-derived polysaccharides have attracted a great deal of attention due to their broad sources and potential bioactivities. *Lentinus edodes*, also known as Xianggu or shiitake mushroom, one of the most popular edible mushroom species due to its unique flavor and high nutritional value, has been widely cultivated and consumed around the world [1]. Polysaccharides extracted from *L. edodes* have been reported to possess several physiological and pharmacological bioactivities including antioxidative, antitumor, antimicrobial and immunomodulatory activities [2]. The polysaccharides obtained from *L. edodes* (LEPs) through water extraction are heteropolysaccharides and mainly consist of glucose, galactose, mannose, xylose and arabinose [3,4]. The physicochemical structure of LEPs varies with the culture medium, extraction process, sites of fruiting body, etc.

Intracellular polysaccharides are usually the main structural feature of the cell wall in fungi [5]. Culture conditions, especially the carbon source, have been found to play an important role in the production and composition of intracellular polysaccharides in fungi, since the carbon source has a direct correlation with cell proliferation and polysaccharide biosynthesis [6]. The carbon source has been reported to affect the activity of key enzymes involved in the polysaccharide biosynthesis pathway, including phosphoglucose-mutase (PGM), UDP-glucose pyrophosphorylase (UGP) and glucosyltransferase (GT) [7]. To elucidate how fungi utilize and metabolize carbon sources, various studies using monosaccharide and disaccharide sources have been performed and have indicated that different species exhibit unique selectivity to the carbon source [6,8]. *L. edodes* is a white-rot fungi that commonly grows on hardwood logs or sawdust by degrading and utilizing lignocellulose, such as hemicellulose, lignin and cellulose. Lignocellulolytic enzymes, including hydrolase and oxidoreductase, as well as potential genes clusters encoding related enzymes, have been identified in *L*. *edodes* [9]. Nowadays, the most common substrate formula for *L. edodes* consists of 78% hardwood sawdust, 18% wheat bran and 2% gypsum, where hardwood sawdust provides the principal energy and carbon source [10]. In order to protect overexploited hardwood resources, several plant-based raw materials and lignocellulosic wastes such as wheat straw, bean stalks and reed grass were investigated as alternative carbon sources for *L. edodes* production [11]. Therefore, it is necessary to investigate the role of lignocellulose components in the growth of *L*. *edodes*. Previous reports have shown that during the initial mycelia growth stage, it is much easier for *L. edodes* to use a more degradable carbon source, such as hemicellulose, rather than lignin and cellulose [12]. However, it was suggested that throughout the growth of *L. edodes* lignin biodegradation is much higher than that of cellulose. To date, the role of lignocellulose in polysaccharide biosynthesis during the mycelia stage has not been studied thoroughly in *L. edodes*. 

Much attention has been drawn to the antioxidative ability of LEPs, as it is a natural, non-toxic compound with fewer side effects. Its effective antioxidant activity has been proven both in vitro and in vivo [13,14]. It has been suggested that the bioactivity of polysaccharides is largely dependent on their chemical composition, molecular weight distribution and other physicochemical properties [15]. Since lignocellulose could affect the physicochemical properties of intracellular polysaccharides, it could also impact the antioxidant properties of LEPs. Our research has showed that hemicellulose could promote the growth of mycelia, while lignin and cellulose had the opposite effect. In order to elucidate the role of lignocellulose in the biosynthesis and characteristics of LEPs, various combinations of hemicellulose and lignin were used as carbon source substitutes for *L. edodes*. The physicochemical properties of LEPs obtained from the culture media were investigated using various methods. Next, the antioxidant properties of LEPs were investigated both in vitro and in vivo. Based on the obtained results, the relationship between the antioxidant activity and the structural characteristics of LEPs was examined.

## 2. Results and Discussion

### 2.1. The Effect of Hemicellulose and Lignin on Mycelial Biomass and LEPs Yield 

**Figure 1:**
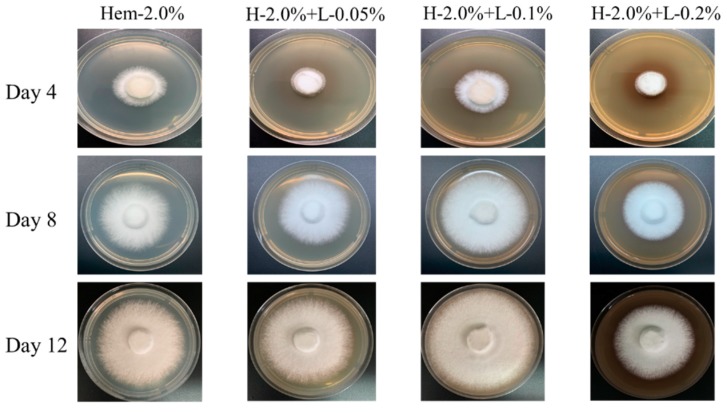
The effect of lignin (0%, 0.05%, 0.1% and 0.2%) combined with 2.0% hemicellulose on the growth of *Lentinus edodes* mycelia. Petri dishes were cultured at 28 °C and photographed every four days until the mycelia of any experimental group had covered the culture medium.

*L. edodes* possesses efficient ligninolytic degradation ability. In addition, lignin has been reported to possess a stimulative effect on mycelia and fruiting bodies of *L. edodes* [16]. In order to examine how lignin affects the utilization of hemicellulose, lignin at different concentrations (0, 0.05, 0.1, 0.2%) combined with 2.0% hemicellulose was used as the synthetic carbon source for *L. edodes*. As shown in Figure 1 and Table 1, 0.1% lignin could stimulate the growth of *L. edodes*. After being cultured for 12 days on H-2.0 + L-0.1%, mycelia covered the petri dish and had an average growth rate of 6.41 mm/day, whereas other samples exhibited lower growth rates. Furthermore, compared to the control group (Hem-2.0% without lignin), 0.2% lignin slightly inhibited the growth of *L. edodes*. This might be the result of cytotoxicity induced by excess lignin. To further analyze the polysaccharide properties and antioxidant activity, 0.1% lignin combined with 2% hemicellulose was used as an experimental carbon source for *L. edodes*. 

To investigate the role of hemicellulose in the growth of mycelia and polysaccharide production in *L. edodes*, various concentrations (2.0, 3.0 and 4.0%, m/v) of hemicellulose were used as a carbon source in a glucose-free potato dextrose liquid medium. Potato dextrose liquid medium containing 2.0% glucose was used as a control. 

**Figure 2:**
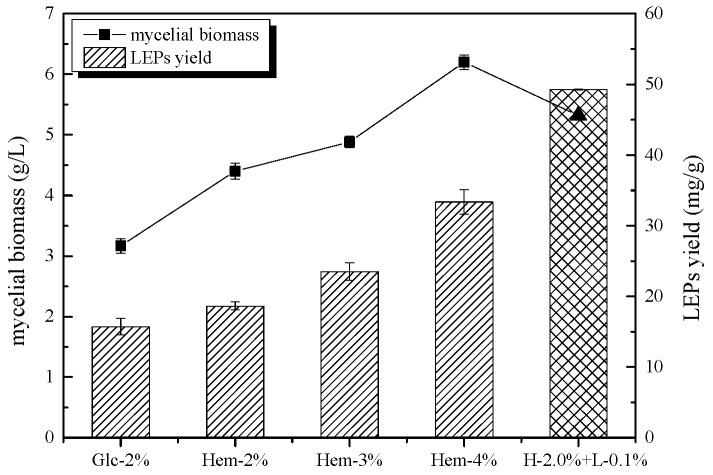
The effect of hemicellulose and lignin on mycelial biomass production and polysaccharide yield in *L. edodes*. The mycelia were cultivated for 10 days at 28 °C with shaking at 160 rpm. *L. edodes* cultivated on potato dextrose medium was used as a control. Values are presented as means ± SD (n ≥ 3).

The mycelial biomass and LEPs production results are shown in Figure 2. The carbon source had a critical effect on mycelial biomass and polysaccharide production. As seen in Figure 2, a higher mycelia biomass yield was found in the media containing hemicellulose. The maximum mycelia yield (6.2 ± 0.1 g/L) was 2.0-fold higher than that of the control group (3.2 ± 0.1 g/L) and was obtained when *L. edodes* was cultured with 4.0% hemicellulose. Moreover, increasing the concentration of hemicellulose from 2.0% to 4.0% led to a gradual increase of mycelia biomass from 4.4 ± 0.1 to 6.2 ± 0.1 g/L. A similar trend was observed when *L. edodes* was cultured with glucose at concentrations ranging from 2% to 4% [17]. The results showed that higher concentrations of hemicellulose induced mycelia growth in *L. edodes*. Furthermore, comparing H-2.0% + L-0.1% treated media with Hem-2% treated media revealed obvious enhancements in mycelial growth (5.3 ± 0.1 g/L as compared to 4.4 ± 0.1 g/L), indicating that an appropriate concentration of lignin could also stimulate the growth of *L. edodes*.

As the mycelium biomass accumulated, the content of polysaccharides that mainly existed in the cell wall and could be extracted and resolved in water increased as well. The effect of hemicellulose and lignin on the production of LEPs was evaluated by comparing the ratio between the polysaccharides and the biomass. As shown in Figure 2, substituting hemicellulose for glucose as the carbon source promoted the biosynthesis of polysaccharides. Specifically, when hemicellulose was at a concentration of 4.0%, polysaccharide production reached up to 2.1-fold higher than that in the control. With increasing hemicellulose concentration, the polysaccharide content was gradually raised from 18.6 ± 0.6 to 33.4 ± 1.7 mg/g (dry mass). Furthermore, the highest production of LEPs (49.3 ± 0.1 mg/g) was observed when hemicellulose was combined with lignin. Compared to the control group, the LEP yield was increased by 213.3%, indicating that lignin (0.1%) either participated in the biosynthesis of LEPs or modified the carbohydrate structure. In addition, lignin was found to increase the expression of genes encoding hemicellulases [18]. Products of lignin degradation by *L. edodes* may act as signaling molecules which participate in the regulation of gene expression involved in sugar transportation and the polysaccharide biosynthesis pathway. These results indicate that polysaccharide production by *L. edodes* is positively correlated with mycelia biomass, as supported by several previous studies [6,19]. Monosaccharides or disaccharides, such as glucose and sucrose, have generally been chosen as the best carbon sources for mycelia biomass and polysaccharide production in various fungi [19,20]. However, our results demonstrated that *L. edodes* Q-7 has a preference for the utilization of hemicellulose as a carbon source. This could be attributed to the different carbon source requirements for mycelial growth and polysaccharide biosynthesis in different species of fungi.

### 2.2. Chemical Analysis of LEPs

To further investigate the chemical composition of LEPs, the total carbohydrate, protein, uronic acid and polyphenol content was measured. As shown in Table 2, H-2.0% + L-0.1% LEP was found to have the highest protein content (2.64 ± 0.17% of polysaccharide), being almost five times higher than that of Glc-2% LEP (0.64 ± 0.18% of polysaccharide). In contrast, the lowest content of uronic acid was found in H-2.0% + L-0.1% treated polysaccharide (5.23 ± 0.72% of polysaccharide), indicating that lignin tends to suppress the generation of uronic acid in *L. edodes*. Among the five polysaccharides tested, the highest content of total polyphenols was seen in H-2.0% + L-0.1% LEP (11.91 ± 0.69 mg GAE/g), whereas the lowest was found in the Glc-2% sample (2.44 ± 0.33 mg GAE/g). In addition, the total protein, uronic acid and polyphenol content of LEPs increased with increasing concentrations of hemicellulose and lignin. Cell wall polysaccharides, especially arabinoxylan, have been reported to be covalently linked to phenolic acids [21] and, upon degradation in the cell, lignin is partially converted to phenol. In addition, the utilization and metabolism of hemicellulose in *L. edodes* might play a role in the biosynthesis of uronic acid, although the mechanism by which this occurs is still not clear. 

### 2.3. Analysis of Monosaccharide Composition

The monosaccharide compositions of LEPs, as determined by a pre-column derivatization HPLC method, are presented in Table 3. LEPs isolated from various *L. edodes* mycelia were identified to be typical heteropolysaccharides, including arabinose, glucose, galactose, xylose and mannose. This illustrates that the monosaccharide composition of LEPs is affected by the culture medium. Xylose, galactose and arabinose were found to be the dominant monosaccharides in all polysaccharide samples. In addition, the, xylose ratio in LEPs increased as the hemicellulose concentration increased. Furthermore, samples grown on media with a hemicellulose–lignin synthetic carbon source produced LEPs with the highest glucose content, suggesting that lignin could promote glucose biosynthesis in *L. edodes*. 

The monosaccharide composition has an essential effect on the physicochemical properties of LEPs. Based on the results shown in Table 3, the type of monosaccharides provided from the culture medium plays a crucial role in determining the monosaccharide composition of LEPs. Xylose, which is degraded from hemicellulose, is utilized by *L. edodes* and participates in the biosynthesis of polysaccharides. Although the mechanism for heteropolysaccharide biosynthesis in *L. edodes* has not been elucidated, it is known that transformations between different monosaccharides occur during the synthesis process [22]. Also, the addition of hemicellulose might promote the conversion of other monosaccharides to xylose. 

### 2.4. Molecular Weight Analysis

**Figure 3:**
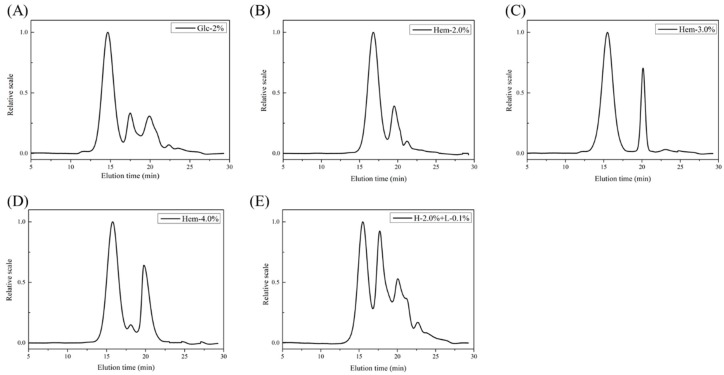
High performance size exclusion chromatography (HPSEC) spectra of polysaccharides obtained from *L. edodes* (LEPs) cultured with different carbon sources. The carbon sources were (**A**) Glc-2.0%, (**B**) Hem-2.0%, (**C**) Hem-3.0%, (**D**) Hem-4.0% and (**E**) H-2.0% + L-0.1%.

Molecular weight plays an important role in the bioactive properties of polysaccharides from *L. edodes*. It has been shown that the degree of branching and polymerization of polysaccharides vary with various carbon sources [6]. The differences in the molecular weight distribution of LEPs cultured using different carbon sources was investigated using high performance size exclusion chromatography coupled with multiple angle laser light scattering and refractive index detector (HPSEC-MALIS-RI). As shown in Figure 3 and Table 4, spectra obtained from polysaccharides extracted from *L. edodes* were mainly composed of two or three peaks, which indicated that the LEPs were polydisperse heteropolysaccharides. The molecular weight distribution of LEPs cultured with hemicellulose tended to be similar and mainly displayed two peaks. However, the ratio of the two polysaccharide components varied with the hemicellulose concentration, suggesting that the addition of hemicellulose led to a lower molecular weight ratio. In addition, when compared to Hem-2.0% LEP, the addition of lignin resulted in a novel polysaccharide component with a molecular weight of 8.42 × 10^6^ Da. Furthermore, the area of Peak1, which had a molecular weight of approximately 20 × 10^6^ Da in Hem-2.0% and H-2.0% + L-0.1% LEPs, occupied 67.83% and 40.36% of the total spectral area respectively. This shows that hemicellulose and lignin could be metabolized and participate in the construction of the cell wall in *L. edodes*. It has been hypothesized that the disaggregation of polysaccharide chains occurs during polysaccharide biosynthesis when *L. edodes* is cultured with lignocellulose. Previous studies have also demonstrated that the Mw of LEPs varies with the concentration of the carbon source in the culture medium [23]. It was hypothesized that lignocellulose, when used as an alternative carbon source, could contribute to the bioactivity of LEPs. 

### 2.5. UV-Visible and FTIR Spectra

**Figure 4:**
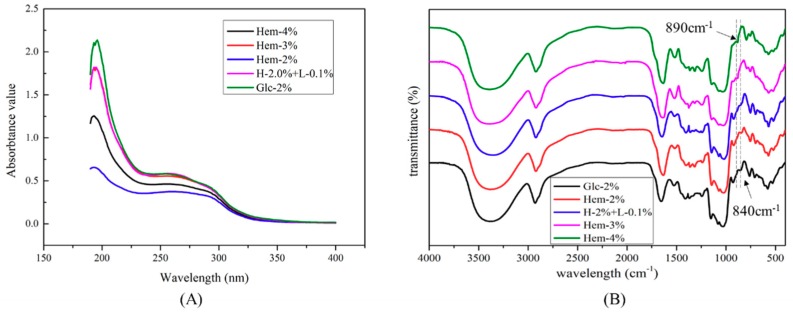
UV-visible and FTIR spectra of LEPs. (**A**) UV-visible spectra were recorded in the range of 190–400 nm. (**B**) FTIR spectra were recorded with a Fourier-transform infrared spectrophotometer (Shimadzu, Japan) between 4000 and 400 cm^−1^.

The UV-visible spectra of the LEPs are depicted in Figure 4A. The absorption peaks at 200–220 nm indicated the presence of polysaccharides. In addition, there was a weak absorption peak observed at 280 nm, suggesting the presence of proteins in the LEPs, which was supported by the chemical measurements. The UV-visible spectra illustrate that there were no significant differences among LEPs derived from various treated mycelia.

FTIR spectra of LEPs from each experimental mycelium were recorded over the frequency range of 4000–400 cm^−1^ (Figure 4B). The various LEPs possessed typical polysaccharide absorption bands. The intense and broad absorption seen at around 3400 cm^−1^ was attributed to the stretching vibration of hydroxyl groups (–OH). C–H stretching vibration was observed at about 2930 cm^−1^ [24]. The asymmetrical peaks at approximately 1650 cm^−1^ and the symmetric peaks around 1410 cm^−1^ were associated with the stretching vibration of deprotonated carboxylic groups [24]. The peaks at approximately 1650 cm^−1^ were also related to the carbonyl groups in the amide group, indicating the existence of residual protein in the LEPs [25]. Furthermore, the absorption at 1080 cm^−1^ was assigned to the stretching vibration of a pyranose ring [26]. The absorption peaks at around 890 cm^−1^ and 840 cm^−1^ were due to the presence of a β-configuration and an α-configuration in the sugar unit, respectively [27]. It was shown that the α- and β-configurations simultaneously exist in the sugar units of LEPs, which were obtained from mycelium cultured with hemicellulose, whereas only the α-configuration was observed in Glc-2% LEPs. Further research will focus on changes to the molecular structure of LEPs affected by lignocellulose.

### 2.6. Antioxidant Activity Assays

#### 2.6.1. Antioxidant Activity In Vitro

**Figure 5:**
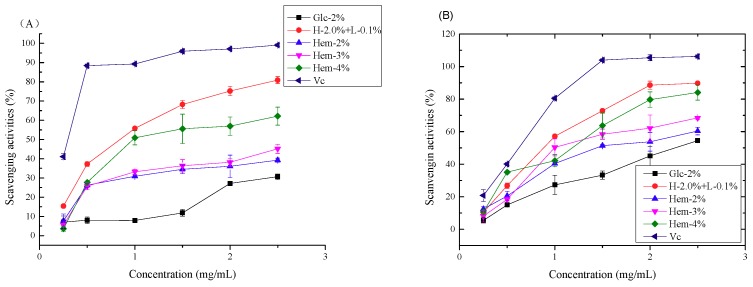

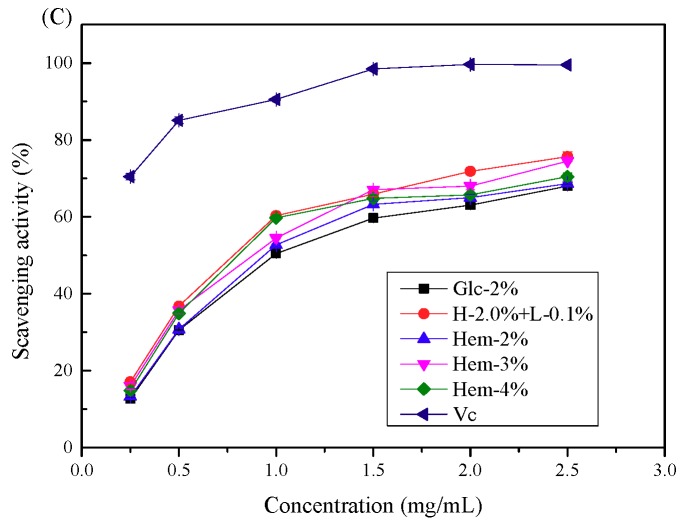
Antioxidant activity of LEPs and ascorbic acid at different concentrations. (**A**) Scavenging activity of LEPs and ascorbic acid on DPPH radicals. (**B**) Scavenging activity of LEPs and ascorbic acid on hydroxyl radicals. (**C**) Scavenging activity of LEPs and ascorbic acid on ABTS radicals. Results are presented as means ± SD (n = 3).

The scavenging activity of LEPs against 1,1-diphenyl-2-picrylhydrazyl (DPPH) radicals was evaluated and is shown Figure 5A. All samples displayed obvious scavenging abilities against DPPH radicals in a concentration-dependent manner within the test concentration range of 0.25 mg/mL to 2.5 mg/mL. In addition, LEPs isolated from *L. edodes* cultured with hemicellulose and lignin exhibited much stronger scavenging abilities in comparison to the control group (Glc-2% LEP). Furthermore, among the polysaccharide samples, H-2.0% + L-0.1% LEP possessed the highest DPPH scavenging activity. At a concentration of 2.5 mg/mL the scavenging abilities of LEPs against DPPH radicals were in the order of: H-2.0% + L-0.1% > Hem-4% > Hem-3% > Hem-2% > Glc-2%, and the scavenging efficiencies were 80.86 ± 1.81%, 62.16 ± 4.69%, 45.17 ± 2.36%, 39.26 ± 1.06% and 30.71 ± 1.44%, respectively. Our results indicated that the hemicellulose concentration is positively correlated with the DPPH scavenging activities of LEPs.

Hydroxyl radicals, which are generated through Fenton reactions in biological cells, are the most reactive among oxygen radical species, and they can attack and damage almost all biomacromolecules [28]. The scavenging activity of LEPs against hydroxyl radicals was evaluated and is shown in Figure 5B. As shown, all polysaccharide samples displayed concentration-dependent scavenging activity on hydroxyl radicals. H-2.0% + L-0.1% LEP showed the best scavenging activity with the lowest IC50 value of 0.81 mg/mL, while the IC50 values of other samples were 2.25 mg/mL, 1.58 mg/mL, 1.21 mg/mL and 0.91 mg/mL for Glc-2%, Hem-2%, Hem-3% and Hem-4%, respectively. Polysaccharides cultured with hemicellulose displayed stronger antioxidant capabilities, which agrees with the results obtained from the DPPH scavenging assay. These results illustrated that the addition of hemicellulose and lignin to the culture medium could promote the antioxidant activity of LEPs.

The 2,2’-azinobis-(3-ethylbenzthiazoline-6-sulphonic acid) (ABTS) radical has been widely used to evaluate the antioxidant potency of natural polysaccharides. As demonstrated in Figure 5C, all of the samples exhibited moderate scavenging activity against the ABTS radical, and the scavenging activity increased with increasing concentrations of LEPs. Among the experimental polysaccharides, H-2.0% + L-0.1% LEP possessed higher ABTS radical scavenging capacity at a concentration of 2.5 mg/mL, while Glc-2% LEP showed the weakest scavenging activity.

Previous studies have illustrated that the antioxidant activity of polysaccharides mainly depends on their physiochemical characteristics, of which monosaccharide composition is one of the most important factors [29]. In the present study, when hemicellulose was employed as the carbon source, LEPs extracted from *L. edodes* had a higher content of xylose than that of Glc-2% LEP, and the xylose content was positively correlated with the hemicellulose concentration, indicating that a higher xylose concentration in polysaccharides contributed to their antioxidant potency. Previously, xylose was reported to play a dominant role in the antioxidant activity of polysaccharides from *Inonotus obliquus* [30]. In addition, a notable increase in the concentration of glucose was found in H-2.0% + L-0.1% LEP, which possessed the strongest scavenging capacity. Thus, it is possible that of the monosaccharides, glucose and xylose are prominent factors associated with the antioxidant activity of LEPs [29].

Together with the results obtained from the chemical composition analysis, the present study also suggested that the antioxidant activity of LEPs is positively correlated with the uronic acid content. H-2%, H-3% and H-4% LEPs with higher uronic acid content displayed higher potency. Previously, it has been demonstrated that the carboxyl groups of uronic acid have a metal ion-chelating ability, which in turn inhibits the generation of hydroxyl radicals [31]. However, H-2.0% + L-0.1% LEP with a relatively lower uronic acid content (5.23 ± 0.72%) exhibited the most effective radical scavenging activity. This suggested that the antioxidant activity of LEPs is also influenced by the total polyphenol and protein content of the polysaccharides. A relatively higher protein and total polyphenol content might result in a higher antioxidant capacity, as has been seen previously [13,15]. In general, the physicochemical characteristics of polysaccharides, including the monosaccharide composition, uronic acid content, protein content and polyphenol content, all contribute to the antioxidant activity. Further investigation is required concerning the purification and deproteinization of polysaccharides. The free radical scavenging mechanism of LEPs also needs to be further explored.

#### 2.6.2. Antioxidant Activity In Vivo

**Figure 6:**
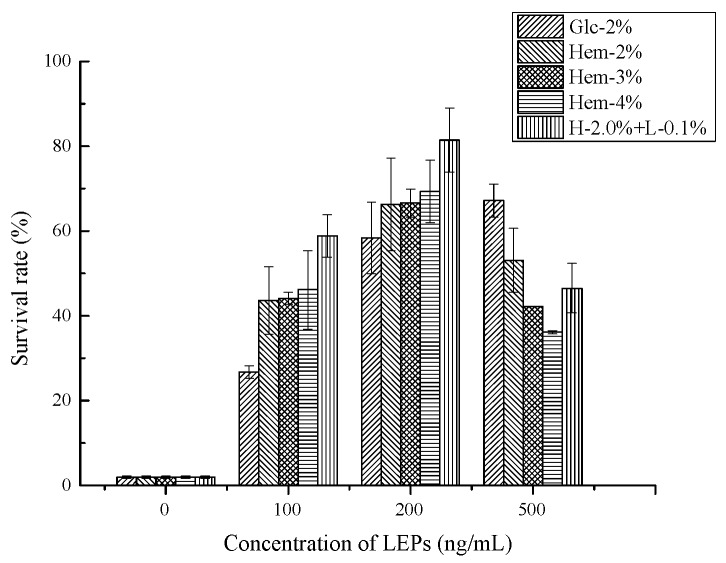
The effect of LEPs on heat shock resistance in *C. elegans*. Thermal tolerance is represented as survival rate when pretreated or untreated *C. elegans* were exposed to 37 °C for 8 h. LEP treatment concentrations ranged from 100 to 500 ng/mL, and the control group was treated with an *E. coli* OP50 suspension mixed with an equal volume of ddH_2_O.

The antioxidant activity of several natural polysaccharides was investigated using *C. elegans* as a model organism, as it shares a wide variety of features with mammals [32,33]. Heat shock is an acute and intrinsic oxidative stress for *C. elegans*. To investigate whether LEPs play a protective role against heat stress, nematodes incubated with LEPs at various concentrations were exposed to heat stress (37 °C) for over 8 h until 95% of the control nematodes died. According to the results depicted in Figure 6, LEP treatment provided protection and increased the survival rate under heat stress. Compared to the control nematodes (not fed with polysaccharides), the treatments containing LEPs at lower concentrations (≤200 ng/mL) exhibited an increasing survival rate in a dose-dependent manner. This indicated that LEPs produce a thermal resistance effect on *C. elegans*. The declining survival rate at an LEP concentration of 500 ng/mL can be explained by the food clearance assay. Nematodes exposed to higher concentrations of polysaccharides might postpone food clearance [34]. Different drugs possessing the optimal dose concentration can be assessed using the *C. elegans* model. Furthermore, compared to the Glc-2% LEP treated nematodes, survival rates were elevated as a result of treatment with Hem-LEPs. The mean survival rate was over 43.6 ± 8.0% in nematodes treated with Hem-LEPs, whereas only 26.7 ± 1.5% of the Glc-2% LEP treated nematodes survived after 37 °C heat stress when the dose was 100 ng/mL. In addition, the survival rate of H-2.0% + L-0.1% LEP treated nematodes was 22.9% higher than that of the Hem-2% LEP treated ones, suggesting that more effective protection against heat shock stress was obtained from polysaccharides cultured with lignin. The in vivo results confirm the antioxidant potential of LEPs, which is in agreement with the results obtained from the in vitro assay.

Heat shock plays a crucial role in the generation and accumulation of hydroxyl radicals, superoxide anion radicals and other reactive oxygen species (ROS) in cells. The elevation of survival rates resulting from LEPs treatments could be attributed to the activation of antioxidant defense mechanisms in *C. elegans*. Previous studies have suggested that the stress response transcription factor DAF-16/FOXO is activated when *C. elegans* is fed with polysaccharides [33]. Moreover, an increase in enzyme activity and a reduction in the lipid peroxidation level were found in the presence of polysaccharides [35]. Accordingly, it is likely that LEPs could trigger the activation of an environmental-stress signaling pathway, therefore causing an upregulation of related genes and alleviating the heat shock damage to *C. elegans*.

## 3. Materials and Methods

### 3.1. Materials and Reagents

*Lentinus edodes* strain Qiuzai No.7 (Q-7) was obtained from Hubei Yuguo Gu ye Co., Ltd. (Suizhou, China). This strain of *L. edodes* was maintained on potato dextrose agar (PDA). The petri plate was incubated at 28 °C for 10 days and then stored at 4 °C.

Monosaccharide standards including glucose, mannose, galactose, xylose, arabinose, rhamnose, ribose and fucose were purchased from Sigma Chemical Co. (St. Louis, MO, USA). Dextran standards with different known molecular weights were purchased from National Institutes for Food and Drug Control (Beijing, China). In this study, hemicellulose and lignin were represented by xylo-oligosaccharide and sodium lignosulfonate, which were purchased from Shandong Longlive Bio-Technology Co., Ltd. (Dezhou, China) and Aladdin Bio-Chem Technology Co., Ltd. (Shanghai, China), respectively. All other reagents used in this study were of analytical grade.

### 3.2. Culture and Experimental Design

Mycelial growth of the shiitake mushroom strain *L. edodes* was activated on a potato dextrose agar (PDA, Beijing Aoboxing Bio-Tech Co., Ltd., Beijing, China) medium in a petri dish at 28 °C for 10 days. For liquid cultures, 10 pieces of fungus blocks (10 mm^2^) were cut from activated PDA media and inoculated into 200 mL of potato dextrose broth (PDB) media. This seed culture was grown at 28 °C with shaking at 160 rpm on a rotary shaker for 10 days and then used to inoculate (10% *v*/*v*) the experimental media. The PDA medium was composed of glucose (20 g) and agar (15 g) in a 1 L potato infusion at pH 6.0 ± 0.1.

In order to evaluate the effect of hemicellulose and lignin on the growth and polysaccharide content of *L. edodes*, hemicellulose was used as the sole carbon source or in combination with lignin as a synthetic carbon source. Potato dextrose liquid medium (2% of glucose) was used as a control. The experimental design is presented in Table 5. All experiments were performed in triplicate at minimum.

### 3.3. Mycelia Preparation and Polysaccharide Extraction

Water-soluble polysaccharides were extracted from the mycelia of *L. edodes* using a previously published method [36]. Briefly, cultured mycelia of *L. edodes* were collected by centrifugation at 8000 rpm for 20 min and washed with distilled water sufficiently, followed by lyophilization. LEPs were obtained by extracting lyophilized mycelia with boiling water for 1 h (1:30, m/v), and then repeating twice. Extraction solutions were gathered and precipitated with four volumes of anhydrous ethanol (1:4, *v*/*v*) at 4 °C overnight to remove oligosaccharides. The resulting mixture was centrifuged at 6000 rpm for 30 min and the supernatant was discarded. The precipitate was washed twice with ethanol and then collected as the polysaccharide of *L. edodes*. Lyophilized LEPs were used for the subsequent analysis.

### 3.4. Characterization of L. edodes Polysaccharides

#### 3.4.1. Determination of Total Carbohydrate, Protein, Uronic Acid and Total Polyphenols Content

The carbohydrate content of the LEPs was measured using the phenol–sulfuric acid method and D-glucose was used as a standard [37]. The protein content of the LEPs was analyzed according to a previously reported method and bovine serum albumin was used as a standard [38]. The uronic acid content was determined by the m-hydroxybiphenyl method and galacturonic acid was used as a standard [39]. The total polyphenol content measurement was performed using the Folin–Ciocalteu colorimetric method and gallic acid was used as a standard. The total polyphenol content was expressed as microgram of gallic acid equivalents (GAE) per gram of dry weight (DW) of polysaccharide.

#### 3.4.2. Determination of Monosaccharide Composition

The monosaccharide composition of LEPs was determined using high-performance liquid chromatography (HPLC) with pre-column derivatization [40]. Briefly, 10 mg of LEPs was first hydrolyzed with 2 mL of 2 M trifluoroacetic acid (TFA) at 105 °C for 9 h. Excess TFA was removed by co-distillation with methanol. Subsequently, hydrolysates of LEPs were derivatized with 1-phenyl-3-methyl-5-pyrazolone (PMP, 0.5 M) at 70 °C for 100 min. The PMP-derivatized samples were run on an Agilent 1260 Infinity HPLC system, coupled with a diode array detector (DAD) and a C_18_ column (4.6 × 250 mm, 5 μm; Agilent, USA). The column was maintained at 30 °C and eluted with a mobile phase consisting of acetonitrile and 0.1 M phosphate buffer (pH 6.7, 17:83, *v*/*v*) at 1.0 mL/min. The wavelength for the DAD was set at 250 nm. The calibration curve was obtained using standard solutions of eight neutral sugars including arabinose, rhamnose, galactose, glucose, mannose, xylose, ribose and fucose.

#### 3.4.3. Determination of Molecular Weight

The molecular weight of the LEPs was determined using high-performance size exclusion chromatography (HPSEC) equipped with a Shodex OHpak SB-806M HQ column (300 × 8 mm), refractive index detector (RI, Waters, Milford, MA, USA) and multi-angle laser light scattering detector (MALLS, DAWN HELEOS-II, 18 angles, Wyatt Technology, Santa Barbara, CA, USA). After filtration through a 0.22 μm membrane, 200 μL of sample solution (1 mg/mL) was injected for each run and eluted with 0.1 M sodium chloride at a flow rate of 0.5 mL/min. Data analysis was performed with ASTRA software (version 6.1.1, Wyatt Technology, Santa Barbara, CA, USA).

#### 3.4.4. UV-Visible Spectra and Fourier-Transform Infrared Spectra

Lyophilized polysaccharide samples were dissolved in ultrapure water and then recorded using a UV-vis spectrophotometer (UV mini-1240, Shimadzu, Kyoto, Japan) using a wavelength range of 190–400 nm. FTIR spectra of LEPs were measured on a Fourier-transform infrared spectrophotometer (Shimadzu, Kyoto, Japan) using a frequency range of 4000–400 cm^−1^. Lyophilized samples were ground with KBr and pressed into a pellet for FTIR determination. A blank KBr pellet was used to collect a background spectrum.

### 3.5. Evaluation of Antioxidant Activity In Vitro

#### 3.5.1. Assay of DPPH Radical Scavenging Activity

The DPPH radical scavenging activity of LEPs samples was determined according to a previously reported method with slight modifications [15]. Briefly, lyophilized polysaccharides were dissolved in distilled water at concentrations of 0.25, 0.5, 1.0, 1.5, 2.0 and 2.5 mg/mL. Next, 120 μL of a freshly prepared 0.1 mM DPPH in ethanol was added to 80 μL of the LEPs samples. Mixtures were shaken and then incubated at 37 °C in the dark for 30 min before being measured at 517 nm on a microplate spectrophotometer (Tecan, Männedorf, Switzerland). Ascorbic acid was used as a positive control and all measurements were taken in triplicate. The scavenging activity of the DPPH radical was calculated according to the following equation:

DPPH radical scavenging activity (%) = [1− (A_2_ − A_1_)/A_0_] × 100 (1)

where A_0_ is the absorbance value of the DPPH-ethanol solution and ultrapure water is used as a negative control, the mixture of sample and ethanol is represented by A_1_ and the mixture of sample and the DPPH-ethanol solution is represented by A_2_.

#### 3.5.2. Assay of Hydroxyl Radical Scavenging Activity

The scavenging capability against hydroxyl radicals was investigated using a Fenton-type reaction according to a method described previously [41]. Briefly, samples were dissolved with distilled water at concentrations ranging from 0.25 to 2.5 mg/mL. Then, 50 μL of LEPs solution was mixed with 25 μL of FeSO_4_ (9 mM) and 50 μL of salicylic acid–ethanol solution (9 mM). Next, 50 μL of hydrogen peroxide (H_2_O_2_, 8.8 mM) was added to start the reaction system. The mixtures were shaken vigorously and then incubated at 37 °C in the dark for 1 h. The absorbance was measured and recorded at 510 nm by a microplate spectrophotometer (Tecan, Männedorf, Switzerland). Measurement steps were repeated for the positive control, which was ascorbic acid. The hydroxyl radical scavenging activity was calculated using the following formula:

Hydroxyl radical scavenging activity (%) = [1 − (B_2_ − B_1_)/B_0_] × 100 (2)

where B_2_ represents the absorbance value of the LEP reaction mixtures and B_1_ and B_0_ are the absorbances of the control with distilled water and a blank control, respectively.

#### 3.5.3. Assay of ABTS Radical Scavenging Activity

The ABTS radical scavenging activity of the LEPs was analyzed according to a previously reported method [40]. Briefly, the ABTS radical solution was generated by mixing 7 mM ABTS solution with a solution of 2.45 mM K_2_S_2_O_8_ for 12 h in the dark at room temperature. Before use, the ABTS^+^ solution was diluted with PBS (0.5 mM, pH 7.4) to an absorbance of 0.60–0.70 at 734 nm. Then, 0.4 mL of sample solution with various concentrations (0.2–2.5 mg/mL) was mixed with 4 mL of ABTS^+^ solution. After reacting in the dark at room temperature for 30 min, the absorbance of the reaction mixture was measured at 734 nm and recorded. Ascorbic acid was used as a positive control. The ABTS radical scavenging ability of LEPs was calculated according to the following equation:

ABTS radical scavenging activity (%) = [1 − (C_2_ − C_1_)/C_0_] × 100 (3)

where C_0_ is the absorbance of the ABTS^+^ solution with ultrapure water, C_1_ is the absorbance of ABTS^+^ solution with different concentrations of polysaccharide sample and C_2_ is the absorbance of the blank (ultrapure water instead of ABTS^+^ solution).

### 3.6. Evaluation of Antioxidant Activity In Vivo

#### 3.6.1. *Caenorhabditis Elegans* Strains, Culture and Synchronization

Wild-type *C. elegans* (N_2_) and uracil mutant *Escherichia coli* OP50 (*E. coli* OP50) were purchased from the *C. elegans* Genetic Center, CGC (University of Minnesota, Minneapolis, MN, USA) and kindly provided by Professor Guangshuo Ou (Tsinghua University, China). *C. elegans* was cultured at 20 °C on nematode growth medium (NGM) with *E. coli* OP50 as the food source, according to a previously published protocol [42]. To obtain synchronized worms, gravid hermaphrodites were treated with 1 mL of freshly prepared lysis buffer (0.6 mL 10% sodium hypochlorite; 1 mL 8 M sodium hydroxide; 3.4 mL ddH_2_O). The released eggs were collected and washed three times using M_9_ buffer (1000 mL aqueous solution containing l5.12 g Na_2_HPO_4_·12H_2_O, 3 g KH_2_PO_4_, 5 g NaCl and 0.25 g MgSO_4_·7H_2_O), then incubated at 20 °C on an NGM plate without food. After 12 h, all of the hatched L_1_ stage larvae were placed on OP50-NGM plates. After synchronization for 28 h, L_4_ stage larvae were transferred to experimental plates.

#### 3.6.2. Heat Shock Stress Assay

The heat shock stress assays were conducted as described previously [43]. Firstly, the age-synchronized L_4_ worms were transferred to experimental NGM plates containing various concentrations of LEPs for 24 h. A plate without LEP was used as a control. The LEPs were diluted from a 50 μg/mL stock solution and added to the *E. coli* OP50 suspension to the final concentrations of 0, 100, 200 and 500 ng/mL and the mixtures were spread on the NGM plates for 6 h at 20 °C. To prevent progeny production, 5-fluoro-2’-deoxy-β-uridine (FUdR; Sigma, St. Louis, MO, USA) was added to a final concentration of 10 μg/mL. After incubation at 20 °C for 24 h, the adults were transferred to an incubator at 37 °C. The surviving nematodes on experimental plates were counted microscopically after 95% of the nematodes in the control group had died. *C. elegans* nematodes were considered dead when they failed to respond to a gentle touch from a platinum wire. Experiments were performed in triplicate with three biological repeats and over 50 worms on each plate.

## 4. Conclusions

In this study, hemicellulose as a carbon source for *L. edodes* growth was shown to significantly increase mycelia biomass and polysaccharide production. The addition of lignin at a concentration of 0.1% prominently stimulated mycelial growth and the synthesis of LEPs. Varying the composition of the carbon source in the culture medium affected the physicochemical structures of LEPs, including the chemical composition, monosaccharide composition and Mw. In particular, the proportion of xylose increased with the increase of hemicellulose, and the biosynthesis of glucose was affected by lignin. Structural analysis, preformed using FTIR, revealed variations between glycosidic linkages in LEPs cultured with hemicellulose. According to free radical scavenging assays, LEPs cultured with hemicellulose and hemicellulose–lignin possessed higher antioxidant bioactivity. Specifically, H-2.0% + L-0.1% treated LEP exhibit the highest antioxidant ability. Furthermore, in vivo experiments performed on *C. elegans* showed that LEPs obtained from a hemicellulose- and lignin-containing culture medium improved the survival rate of nematodes under thermal stress. In summary, we demonstrated that hemicellulose and lignin can enhance the yield of *L. edodes* as well as influence the physicochemical properties and promote the antioxidant activity of LEPs. However, the role of hemicellulose and lignin in the *L. edodes* polysaccharide biosynthesis pathway merits further investigation. We believe that the presented results will provide a strategy for *L. edodes* cultivation and polysaccharide production.

## Figures and Tables

**Table 1:** The effect of lignin (0%, 0.05%, 0.1% and 0.2%) combined with 2.0% hemicellulose on the mycelial growth rate of *L. edodes*.

Samples	Mycelium Colony Diameter (mm)				Average Growth Rate (mm/day)
	Day 1	Day 4	Day 8	Day 12	
Hem-2.0%	10 ± 0.03	24 ± 0.03	47 ± 0.12	59 ± 0.06	4.08
H-2.0% + L-0.05%	10 ± 0.06	19 ± 0.11	47 ± 0.16	62 ± 0.1	4.33
H-2.0% + L-0.1%	10 ± 0.06	25 ± 0.55	60 ± 0.54	87 ± 0.21	6.41
H-2.0% + L-0.2%	10 ± 0.01	15 ± 0.11	39 ± 0.15	48 ± 0.17	3.16

**Table 2:** Chemical composition analysis of polysaccharides obtained from *L. edodes*. ^1^

Samples	Protein (%)	Uronic Acid (%)	Total Phenolics (mg GAE/g) ^2^
Glc-2%	0.64 ± 0.18	6.77 ± 0.27	2.44 ± 0.33
Hem-2%	1.03 ± 0.13	6.88 ± 0.31	7.39 ± 0.21
Hem-3%	1.42 ± 0.05	7.49 ± 0.08	7.48 ± 0.26
Hem-4%	1.80 ± 0.08	7.54 ± 0.22	10.90 ± 0.91
H-2.0% + L-0.1%	2.64 ± 0.17	5.23±0.72	11.91 ± 0.69

^1^ Values are expressed as means ± SD. (n = 3). ^2^ GAE represented gallic acid equivalents.

**Table 3:** Monosaccharide composition of the polysaccharides extracted from *L. edodes* cultured with different concentrations of hemicellulose and lignin. ^1^

Samples	Ara (%)	Glc (%)	Xyl (%)	Gal (%)	Man (%)
Glc-2%	19.49	15.79	15.79	39.18	9.75
Hem-2%	21.14	15.64	25.16	32.77	5.29
Hem-3%	23.47	11.74	30.28	29.58	4.93
Hem-4%	23.04	13.59	35.94	23.27	4.15
Hem-2.0% + L-0.1%	18.69	26.36	17.01	31.78	6.17

^1^ Analyzed by high-performance liquid chromatography equipped with a diode array detector (HPLC-DAD), after acid hydrolysis and 1-phenyl-3-methyl-5-pyrazolone (PMP) pre-column derivatization. Ara: arabinose; Glu: glucose; Gal: galactose; Xyl: xylose; Man: mannose.

**Table 4:** Molecular weight distribution of polysaccharides extracted from *L. edodes* cultured with different concentrations of hemicellulose and lignin (10^3^ KDa). ^1^

Samples	Peak1				Peak2				Peak3			
	Mw	Mn	Mw/Mn	Ratio (%)	Mw	Mn	Mw/Mn	Ratio (%)	Mw	Mn	Mw/Mn	Ratio (%)
Glc-2%	35.43	30.54	1.16	64.20	12.05	10.82	1.11	17.06	1.31	1.16	1.117	18.74
Hem-2%	16.36	13.3	1.23	67.83	-	-	-	-	1.58	1.23	1.28	32.17
Hem-3%	21.47	15.9	1.35	77.34	-	-	-	-	1.00	0.94	1.06	22.66
Hem-4%	19.63	16.36	1.20	63.18	-	-	-	-	1.33	1.17	1.14	36.82
H-2.0% + L-0.1%	20.71	17.7	1.17	40.36	8.42	7.32	1.15	34.89	0.92	0.63	1.46	24.75

^1^ Determined by high-performance size exclusion chromatography coupled with refractive index detector (RI) and multi-angle laser light scattering detector (HPSEC-MALIS-RI). Mw: molecular weight; Mn: number-average molecular weight. “-”: not detected.

**Table 5:** Experimental design of this study.

Carbon source	Concentration (m/v)	Abbreviation	pH
hemicellulose	2%, 3%, 4%	Hem-2%, 3%, 4%	6
hemicellulose + lignin	2.0% + 0.05%	H-2.0% + L-0.05%	
	2.0% + 0.1%	H-2.0% + L-0.1%	
	2.0% + 0.2%	H-2.0% + L-0.2%	
glucose	2%	Glc-2%	
